# Microporous Hyper-Crosslinked Polystyrenes and Nanocomposites with High Adsorption Properties: A Review

**DOI:** 10.3390/polym9120651

**Published:** 2017-11-28

**Authors:** Rachele Castaldo, Gennaro Gentile, Maurizio Avella, Cosimo Carfagna, Veronica Ambrogi

**Affiliations:** 1 Institute for Polymers, Composites and Biomaterials, National Research Council of Italy, Via Campi Flegrei 34, 80078 Pozzuoli, Italy; gennaro.gentile@cnr.it (G.G.); maurizio.avella@cnr.it (M.A.); carfagna@unina.it (C.C.); 2 Department of Chemical, Materials and Production Engineering, University of Naples, Piazzale Tecchio 80, 80125 Napoli, Italy; ambrogi@unina.it

**Keywords:** hyper-crosslinked polystyrene, hyper-crosslinked nanocomposites, microporous materials, high specific surface area, adsorption properties

## Abstract

Hyper-crosslinked (HCL) polystyrenes show outstanding properties, such as high specific surface area and adsorption capability. Several researches have been recently focused on tailoring their performance for specific applications, such as gas adsorption and separation, energy storage, air and water purification processes, and catalysis. In this review, main strategies for the realization of HCL polystyrene-based materials with advanced properties are reported, including a summary of the synthetic routes that are adopted for their realization and the chemical modification approaches that are used to impart them specific functionalities. Moreover, the most up to date results on the synthesis of HCL polystyrene-based nanocomposites that are realized by embedding these high surface area polymers with metal, metal oxide, and carbon-based nanofillers are discussed in detail, underlining the high potential applicability of these systems in different fields.

## 1. Introduction

Porous materials have always attracted high scientific interest because of their outstanding performance and potential applications in various fields, such as gas adsorption and separation [1,2], energy storage [3], air and water purification processes [4,5,6], drug delivery [7,8,9], molecular separation [10,11], catalysis [12,13], sensing [14], optoelectronics [15], and preparation of superhydrophobic surfaces [16].

Several properties of the bulk materials depend on their porosity, and huge opportunities are offered today by the possibility of tailoring different porous structures. In particular, porous materials display extremely high specific surface area (SSA) and outstanding adsorption properties if nanosized porosity is present in a significant amount. Pores are classified based on their average size, by the International Union of Pure and Applied Chemistry (IUPAC), in micropores if the pores internal width is below 2 nm, mesopores if it is between 2 and 50 nm, and macropores if the internal width is over 50 nm [17]. Moreover, pores can be open or closed, slit-shaped, i.e., characterized by plane walls, or cylindrical, and the whole porosity can be composed of an ordered network or a disordered set of voids. Crystalline materials like zeolites [18] and metal-organic frameworks [19], for example, possess a regular and well defined system of pores, while amorphous materials, like activated carbons [20] and different classes of polymers [21,22,23], possess an interconnected irregular-shaped porosity.

Hyper-crosslinked (HCL) polymers are a class of microporous polymers that are prepared by the extensive crosslinking of linear or lightly crosslinked precursor polymers. They can be obtained by a two-step or by a one-pot process, starting from several aromatic monomers [24,25,26,27,28]. Hyper-crosslinked polymers (HCP) show extremely high surface areas and porosity, low density, outstanding adsorption properties, high chemical, and thermal stability [29].

The first example of HCP, synthesised by Davankov and co-workers, was based on polystyrene (HCL PS) [30]. Since then, many researches have focused on HCL PS-based polymers, diversifying, for example, the crosslinking degree of the precursor polymer in order to modulate the HCL PS pore size distribution and SSA (up to ~2000 m^2^/g). In addition, various synthetic procedures were explored to obtain highly crosslinked materials in different morphologies. Moreover, the introduction of functionalized monomers or the post-crosslinking chemical modification were considered to impart specific functionality to the HCL PS.

Other classes of hyper-crosslinked polymers have been explored more recently. Polysulfone, for example, was hyper-crosslinked through a two-step reaction, consisting into the grafting of a bromomethyl moiety onto the polysulfone benzene rings and the successive hyper-crosslinking through Friedel-Crafts reaction. However, polysulfone shows low activity in the Friedel-Crafts reaction and a high inherent chain flexibility, which both limit the achieving of a high hyper-crosslinking degree, leading to low SSA (~70 m^2^/g) materials [31]. Hyper-crosslinking of polyaniline through a microwave assisted process, instead, allowed for obtaining a material with an SSA of about 1000 m^2^/g and a high adsorption capability towards CO_2_ and H_2_ [32]. A natural polymer like lignin was also crosslinked by using an external crosslinker, formaldehyde dimethyl acetal (FDA), and, upon a pyrolysis treatment, this material displayed SSA of about 100 m^2^/g and high CO_2_/N_2_ selectivity [33].

Nevertheless, when considering their possible wide differentiation in terms of monomer composition, pre- or post-functionalization, and the type of synthetic approach adopted, HCL PS are still the most representative class of high SSA materials. Furthermore, recent research works have demonstrated the possibility of tuning specific functional properties of HCL PS resins, such gas sorption properties, including gas sorption selectivity, and the capability to sequester organic pollutants, by way of adding various functional nanostructured materials [34]. This approach has opened the way to the realization of a new class of very high performances microporous hyper-crosslinked nanocomposites.

In this review, after summarizing major chemical strategies to synthesize microporous HCL PS resins, we will describe the progresses obtained on the realization of HCL nanocomposites, with a view to evaluating their potentiality for applications in different sectors.

## 2. HCL Polystyrenes

One of the first approaches to obtain highly porous PS-based polymers consisted on the polymerization of a copolymer obtained by styrene (ST) and a bifunctional monomer, such as divinylbenzene (DVB), in the presence of a porogenic agent. During polymerization, the bi-functionality of the DVB is responsible of the formation of crosslinks between the polymeric chains. The removal of the porogenic agent leaves a porous structure, which can display specific surface area (SSA) from 50 to 1000 m^2^/g [35]. In this way, high surface area structures are usually obtained by using high amounts of the bifunctional monomer (DVB > 50 mol %). However, in this polymerization process, as the reaction proceeds, DVB also induces steric congestion, preventing the proceeding of the reaction. As a result, many vinyl groups remain unreacted at the end of the reaction and this limitation in the extent of crosslinking is practically unavoidable.

**Figure 1:**
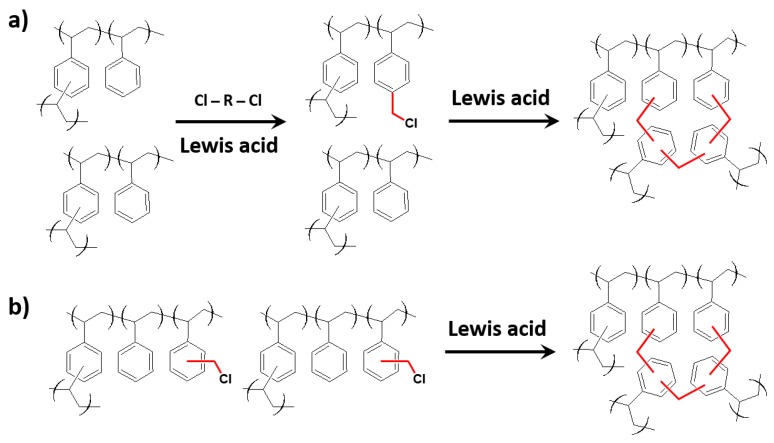
Hyper-crosslinking reaction scheme through external (**a**) and internal (**b**) electrophile.

In 1969, Davankov and co-workers introduced a new method for the production of very highly porous PS-based polymers, obtained by crosslinking PS in a highly swollen state by an external crosslinker [30]. The crosslinker proposed is a bifunctional electrophilic compound that, upon activation by a Friedel-Crafts catalyst, installs structural bridges between the PS aromatic rings. The use of an external crosslinker permits to overcome the steric congestion limitation, thus creating a highly interconnected three-dimensional network, which is characterized by spatially non-planar cycles that are formed by covalent bridges and very short segments that are confined between the branching points (see Figure 1a). This network porosity remains also after solvent removal, conferring the material high specific surface area and micropore volume. This procedure was further developed, first by Veverka and Jerábek [36] and then by Ahn et al. [37], who proposed the use of an internal electrophile, more specifically a reactive comonomer, vinylbenzyl chloride (VBC). The chloromethyl groups of VBC, upon activation by a Friedel-Crafts catalyst, are able to create methylene bridges between neighboring aromatic rings (see Figure 1b).

In both cases, the polymers acquire a highly crosslinked structure, and, therefore, they are well-known as hyper-crosslinked polystyrenes, also called “Davankov-type resins” [38].

### 2.1. Precursor Polymers

**Figure 2:**
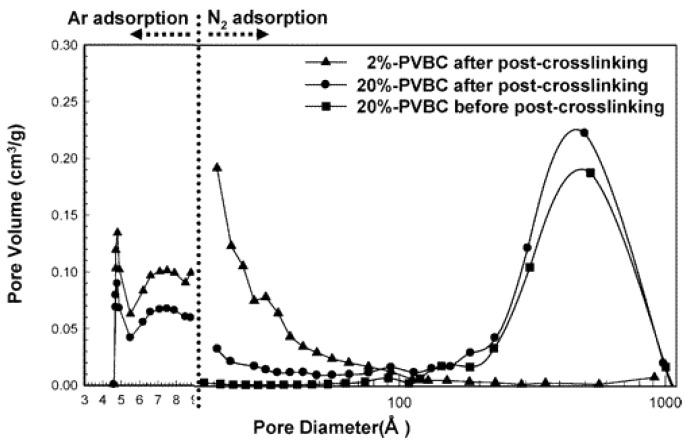
Pore size distribution of divinylbenzene/vinylbenzyl chloride polymers containing 2 and 20 mol % DVB. Reprinted with permission from reference [37]. Copyright 2006 American Chemical Society (Washington, United States of America).

The effect of the precursor polymers on the final HCP was extensively studied by many research groups. Ahn et al. [37], for example, explored the effect of the monomer composition in DVB/ST/VBC polymers obtained by suspension polymerization and successive hyper-crosslinking. Ascertaining that using higher amounts of DVB leads to high specific surface areas HCP, they assessed that the DVB content had an important impact on the pore size distribution of the obtained materials. In fact, for low DVB content, such as ~2 mol %, the obtained hyper-crosslinked polymers were characterized by major microporosity, while higher DVB inclusion, such as ~20%, led to bimodally distributed porosity, with a major mesoporosity peak and a minor microporosity one. In the latter case, in fact, mesoporosity is created by DVB crosslinks in the polymerization step, while microporosity is formed during the hyper-crosslinked step. In this way, a lower surface area material is obtained, with BET SSA reaching maximum 1000 m^2^/g, while in the first case (DVB ~2 mol %), higher surface area polymers are obtained, with BET SSA up to 2000 m^2^/g. The pore size distribution of DVB/VBC polymers containing 2 and 20 mol % DVB are reported in Figure 2, where microporosity data are evaluated by Ar adsorption porosimetry for pores diameters below 9 Å and by N_2_ adsorption for pores from 10 to 1000 Å. Argon is generally considered more suitable than nitrogen for pore size measurements of ultramicropores (below 0.7 nm), since Ar fills ultramicropores at a much higher relative pressure with respect to nitrogen, in conditions where the gas diffusion is quicker, leading to faster equilibration processes and more accurate analyses [39].

The effect of the use of VBC as an internal crosslinker was also extensively studied [37]. The Friedel-Crafts reaction employing internal electrophiles (in this case VBC) is extremely efficient. The reaction was found to reach almost completion after 15 min (as determined by the steep decrease in Cl content from ~19% to ~2% and SSA increase from ~0 to 1200 m^2^/g) and achieve conclusion after 2 h, when the values of SSA and residual Cl reached a plateau, with SSA = 1800 m^2^/g and Cl ~2%. They suggested that the internal electrophile is so efficient because the last step of the reaction is particularly favorable, being the aromatic ring to be substituted is already doubly alkylated, and thus, electron rich. Fontanals et al. [40] tested the effects of different VBC isomers on HCP obtained by suspension polymerization and successive hyper-crosslinking. They found that polymers that were prepared by *p*-VBC (para-VBC) and mix-VBC (mixture of 70:30 = meta:para VBC isomers) differed in the residual chlorine content, mainly due to preferential hydrolysis of *p*-VBC during the suspension polymerization. They reported that the hydrolysis of the VBC chloromethyl group was expected to proceed through a SN1 mechanism, which involved the formation of a stabilized carbocation intermediate, followed by nucleophilic attack of water. Therefore, since *p*-VBC forms a more stable carbocation with respect to *m*-VBC, this translates into a more favorable hydrolysis for *p*-VBC. As a result, the HCP based on *p*-VBC or mix-VBC showed, respectively, a higher hydrophilic character or higher SSA. When comparing a *p*-VBC-derived HCP and a mix-VBC-derived HCP, both containing 2 mol % DVB and 98 mol % VBC, they found that the first one contained 4 wt % of oxygen and displayed 900 m^2^/g BET SSA, and the second had 1.5 wt % of oxygen and a 1900 m^2^/g BET SSA. 

Another approach to the choice of the proper precursors is oriented to the adsorption properties of the HCP. Germain et al. [41,42], for example, investigated the effect of polyaniline and polypyrrole as precursors on the H_2_ adsorption capacity of the hyper-crosslinked resin. Polyaniline was previously reported as a potential H_2_ storage material, but they found that the protonation of HCP containing polyaniline diminishes the polyaniline ability to adsorb hydrogen. Indeed, while the aniline functionality is electron-donating and favours the H_2_ adsorption, protonated aniline, which is highly electron-withdrawing, decreases it. Polypyrrole, then, was found, through various synthetic techniques, to lead to higher H_2_-polymer interactions when introduced in HCP. Dawson et al. [43], on the other hand, prepared a series of HCP based on benzene and aniline in different relative ratios to enhance the CO_2_ adsorption capacity of the HCL resin, since aniline was reported to adsorb preferentially CO_2_. They found that SSA decreased as the aniline amount in the polymer increased, as expected, and a good compromise was found when aniline was included as the 40 mol % (SSA ~200 m^2^/g and CO_2_ uptake ~1.2 mmol/g). Furthermore, the difference between the SSA and the CO_2_ decreasing rates suggested that these materials could have good selectivity for CO_2_/N_2_ adsorption.

### 2.2. Synthetic Routes

Davankov synthesised the first HCP starting from polystyrene that was dissolved in an organic medium (up to 10–11% *w*/*v*) in the presence of a crosslinker and a Friedel-Crafts catalyst. The crosslinking agents were bifunctional aromatic acyl or alkyl halides, and the media were several organic solvents that were able to dissolve PS without deactivating the Friedel-Crafts catalyst. During hyper-crosslinking, the solution was rapidly transformed into a gel block, which eventually shrinked and crushed over the completion of the reaction. Thus, the resulting HCL polymer resulted in the form of rigid particles of an irregular shape. Then, DVB was included as a comonomer of ST, in the range 0.3–2 mol %, and this permitted also to obtain precursor copolymers in the form of spherical gel-type beads. In this case, the reaction medium was any solvent that was able to swell the precursor copolymer [30,44]. The beads preparation was further developed by many research groups. Beads were obtained by suspension polymerization, usually leading to polydisperse particles in the range 10–500 µm, which showed morphological changes during the Friedel-Crafts reaction. Emulsion polymerization, on the other hand, allowed for obtaining smaller and more homogeneous particles, as Macintyre et al. [45] demonstrated by preparing near monodisperse beads with 420 nm medium diameter, able to preserve their shape even after hyper-crosslinking. Non-aqueous dispersion polymerization (NAD) and precipitation polymerization (PP) were also employed to obtain uniform particle size, giving, respectively, beads with 4–10 µm diameter, and monodisperse 4 µm beads. Apart from size and shape, the precursor polymerization synthesis was also found to influence the HCP final SSA and porosity. In particular, for NAD and PP, an amount of VBC higher than 50 mol % was required to obtain HCP with SSA higher than 1000 m^2^/g [46]. In contrast, for suspension polymerization, 20 mol % of VBC monomer was sufficient to obtain HCP with SSA over 1000 m^2^/g. The highest surface area HCP, as obtained by suspension polymerization, showed SSA up to 2090 m^2^/g and it was prepared using DVB, ST and VBC in the molar ratio DVB:ST:VBC = 2:0:98 [37].

Another synthetic approach was proposed by Fréchet and Svec [10], who prepared DVB/ST/VBC based HCP in the form of monolith performing in situ hyper-crosslinking in capillary columns in the presence of organic porogens (toluene and 1-dodecanol), obtaining a material with SSA of 663 m^2^/g. In another work, Tong et al. [47] compared monolithic and bead-shaped DVB/VBC hyper-crosslinked polymers for methane storage, evidencing the advantage of avoiding packing problems, which are typical of particulate materials. In their case, the hyper-crosslinked monolith and beads exhibited roughly similar porous and adsorption properties, but the monolith display a 116% higher methane storage capacity for equal volume.

A further synthetic route that pursued to obtain high surface area hyper-crosslinked polymers is the self-condensation of benzyl chloride containing monomers. Tsyurupa and Davankov [48], in 2002, first reported the polycondensation of *p*-dichloroxylene (*p*-DCX) in presence of SnCl_4_. Chaikittisilp et al. [49] prepared HCP that was characterized by SSA of ~2500 m^2^/g, using benzyl chloride terminated double-four-ring cubic siloxane cages as precursors. This particular procedure was based on the contemporary polymerization of the benzyl chloride terminations and the destruction of the siloxane cages, and led to obtaining a highly porous material that was characterized by hierarchical micro/meso porosity. Yuan et al. [50] prepared high surface area polymers from self-condensation of carboranes that were previously functionalized by the simple grafting of two benzyl chloride groups. They obtained HCP with a surface area up to 1023 m^2^/g and relatively high H_2_ adsorption, which was presumably ascribable to the strong electrophilicity of the highly electronically deficient carborane units.

### 2.3. External Crosslinkers, Catalysts and Solvents

**Figure 3:**
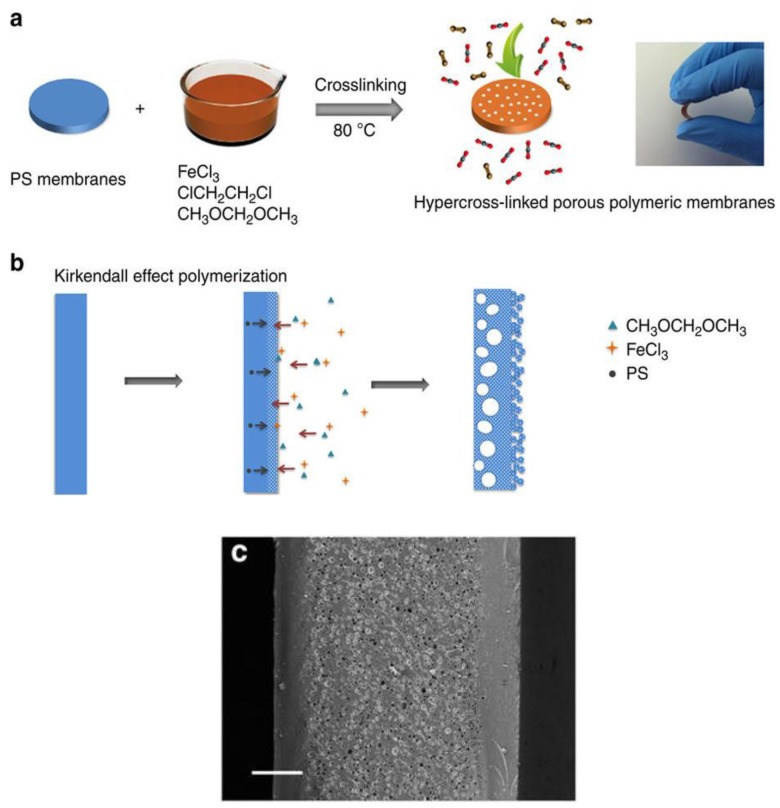
Synthesis of the hyper-crosslinked porous polymeric membranes. (**a**) Schematic illustration of the preparation procedure for the porous polymeric membranes; (**b**) the non-equilibrium diffusion at the interface (the reaction is only shown for one side of the membrane); and, (**c**) SEM images of the cross-section of the porous polymeric membranes crosslinked for 24 h (scale bar 20 μm). Reprinted by permission from Macmillan Publishers Ltd. (Basingstoke, UK): Nature Communications [57], copyright 2014.

The first external crosslinker that was used in the hyper-crosslinking step was chloromethyl ether. Other reagents, such as carbon tetrachloride, monochlorodimethyl ether, dichloroxylene, or dichloroethane, which can act both as an external crosslinker and a solvent, were also reported [44,51,52,53,54,55]. More recently, Li et al. [56] proposed the use of formaldehyde dimethyl acetal, which gives methanol as a by-product instead of chlorinated compounds. The procedure proposed allowed for preparing HCP in a one-step procedure, dissolving the aromatic monomers, FDA, and the Friedel-Crafts catalyst in the proper solvent. By adjusting the type of monomers and the amount of FDA, the largest SSA value achieved was ~1400 m^2^/g, for a FDA:benzene molar ratio of 3:1. Through the knitting technique, Dai and co-workers [57] obtained high surface area flexible polystyrene membranes, starting from commercial PS membranes, using FDA as a crosslinker and FeCl_3_ as a catalyst. Their procedure was based on the evidence that the external layers of the PS membranes first react very fast with FDA, producing a microporous insoluble shell, and then hyper-crosslinking proceeds inside the membrane, while part of the PS dissolves and moves from the core to the shell, finally creating a sandwich-structured polymeric molecular sieve membrane with a hierarchical and tailorable porosity (see Figure 3). The hyper-crosslinked PS membranes displayed specific surface areas from 260 to 792 m^2^/g, increasing as the membrane thickness decreased from 109 to 86 µm. In fact, as the hyper-crosslinking process begins from the outside of the membrane, it proceeds more easily on thinner ones, due to a faster mass transport, resulting in higher specific surface area. Moreover, all of the membranes showed hierarchical porosity and predominant microporosity. In particular, they showed similar pore size distribution, with a dominant pore size of about 5 Å, and mesopores size in the range 2–4.4 nm.

Another type of hyper-crosslinked PS membrane was prepared by Tan and co-workers [58]. They obtained soluble HCP by inducing intramolecular crosslinking of PS. They exploited the fact that at very low concentrations the polystyrene precursor is highly dispersed and expanded, which favours the intramolecular crosslinking with respect to intermolecular knitting. This soluble HCP showed relatively lower surface area but, on the other hand, was solution-processable to produce thin hyper-crosslinked membranes.

Friedel-Crafts catalysts that are suitable for the hyper-crosslinking reactions are Lewis acids or protonic acids. In principle, all of the Lewis acids could be used, but poor solubility and steric hindrance of catalysts are some factors that can limit an extensive hyper-crosslinking. The latter is a particularly relevant issue when considering that, after the first crosslinks form, larger catalysts may be obstructed from accessing the chloromethyl moieties and activate the formation of further crosslinks [59].

Hyper-crosslinking is typically performed in a thermodynamically favourable solvent for the polymer, in which the precursor resin may dissolve or swell, depending on the linear or lightly crosslinked structure. The extensive crosslinking locks the polymer chains while they are in an expanded form, preventing them from collapsing after solvent removal, thus creating a three-dimensional structure that is made of interconnected pores. Dichloroethane (DCE) is one of the most widely used solvents for hyper-crosslinking, although various solvents have been experimented, such as nitrobenzene, chlorobenzene, cyclohexane, hexane, and different mixtures of them. Surprisingly, also bad solvent for PS, such as hexane and chlorobenzene, are effective in the reaction leading to high surface area HCP [30,44]. Moreover, the HCP are able to swell in a broad spectrum of solvents, regardless of their thermodynamic affinity with the precursor polymer. For example, despite their hydrophobicity, PS-based HCP are able to adsorb almost at the same level polar protic solvents, such as water and methanol, and good solvents for the precursor polymers, such as toluene and THF [37]. The swelling behavior of crosslinked polymers is still well described by the Flory-Rehner theory, which assumes the additivity and independence of the thermodynamic effects at the basis of the solvent uptake, as described by the Flory-Huggins theory, and the elastic restoring force of the polymer network [60]. Relationships between swelling and porosity in crosslinked copolymer networks, including styrene based porous systems, have been well discussed by Okay [61].

### 2.4. Crosslinking Degree

Inner molecular structure plays a key role for the physical properties of the HCL-materials. Davankov and Tsyurupa [62] explored the effect of the degree of crosslinking on the structure and adsorption activity of the HCP. Styrene/divinylbenzene (0.5 mol % DVB) polymers were crosslinked with monochlorodimethyl ether (MCDE), in presence of a Friedel-Crafts catalyst, at different crosslinking degrees. By using from 0.3 mol of MCDE per mole of PS to 2.5 mol MCDE/mol PS, HCP characterized by crosslinking degree from 43 to 500% were obtained, where 100% crosslinking degree means that each phenyl ring participates in the formation of a chloromethyl bridge. They found that 100% crosslinking was obtained with 0.5 moles of crosslinker per mole of PS. In this way, HCP with SSA in the range 1300–1500 m^2^/g were obtained for crosslinking degrees from 43 to 400%, and SSA of about 1000 m^2^/g was registered for 500% crosslinking. The highest pore volume and swelling degree was found for 300% crosslinking. The adsorption of dyes from aqueous solution increased with the degree of network swelling in water, reaching the maximum for 300% crosslinking, thus resulting to be driven more from the ability of the HCP to swell in water than from the inner SSA of the dry sample.

One of the most peculiar characteristics of the HCP is the capacity to swell in any kind of solvent, and this phenomenon is strictly connected to the highly crosslinked structure of the material. Highly crosslinked HCP possess inner stresses that are developed during hyper-crosslinking and solvent removal. These stresses release by swelling when the dry polymer adsorbs a solvent, and therefore, even if the solvent interaction with the polymer is weak, the swelling state is more energetically favourable than the dry one [48].

A better understanding of the HCP structure and porosity formation, in order to better design this type of polymers for appropriate applications, can be provided from molecular simulation. When considering the complexity of these highly crosslinked amorphous materials, molecular simulation does not provide the maximum accuracy for these systems, but it can still be a reliable complement to instrumental characterization. The simulation process permits to monitor the evolution of the pore networks through the polymerization, the hyper-crosslinking, and the final relaxation of the network [63,64,65,66].

### 2.5. Chemical Modification

Sorption properties of polystyrene-based hyper-crosslinked polymers basically rely on hydrophobic or π–π interactions. One of the advantages of HCP, as compared to other high surface area materials, is their easy chemical modification, either before or after the hyper-crosslinking process, which permits to widely broaden their potential application in adsorption.

For VBC-based polymers, a very efficient and practical functionalization strategy is to exploit the residual chloromethyl groups after hyper-crosslinking. Li et al. [67] followed this route to modify HCP with dimethylamine, obtaining amine modified HCP with enhanced adsorption properties towards polar compounds. Cormack et al. [68] adopted this strategy using acetyl sulfate or lauroyl sulfate to produce sulfonated HCP and improve the resin ion-exchange properties; also, an HCP synthesised from Friedel-Crafts alkylation between carbazole and α,α′-dibromo *p*-xylene was subjected to modification via sulfonation and transformed in a very efficient material for solid acid catalysis for the production of biodiesel [69].

**Figure 4:**
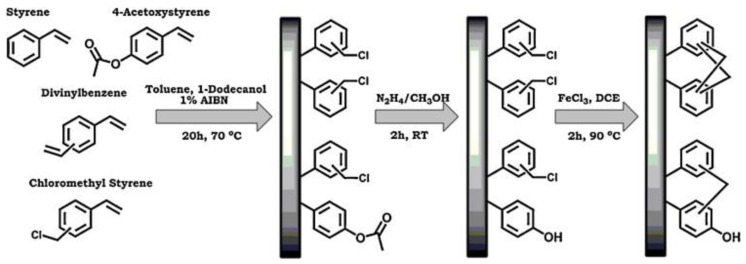
Schematic representation of the preparation of hyper-crosslinked monoliths with phenol functionalities using copolymerization of 4-acetoxystyrene, followed by deprotection and hyper-crosslinking. Reprinted from ref. [70], Copyright 2013, with permission from Elsevier (Amsterdam, The Netherlands).

Another very interesting approach to optimize HCP capillary monoliths efficiency with proper functionalization was proposed by Maya and Svec [70]. Their approach consisted in introducing a monomer with a protected functionality in the polymerization mixture, and then deprotect it in a second step. Specifically, a 4-acetoxystyrene was copolymerized with DVB, ST, and VBC to produce monolithic columns. Deprotection of the functionality converted the acetoxy functionalities to phenolic hydroxyl groups, thus changing the polarity of the HCP (see Figure 4). This technique results in monoliths with tunable chemistry, depending on the extent of the deprotection reaction, and is based on a simple principle that is valid for a wide range of functionalization.

## 3. HCL Polystyrene Based Nanocomposites

HCL microporous nanocomposites are nowadays a class of materials that are widely studied for applications in adsorption and catalysis [71,72].

As soon as it was discovered, the microporous structure of HCL PS-based resins was immediately considered as potentially interesting to guest nanoparticles with specific functionalities [73]. Different approaches were used to embed nanoparticles within the matrix nanocavities, either based on the post-growth of nanostructured particles on the hyper-crosslinked PS matrix, or, more recently, by direct embedding of pre-formed nanoparticles during the pre-polymerization step, followed by the hyper-crosslinking. Nanostructured HCL PS-based nanocomposites obtained up to now can be mainly categorised, based on the nature of the nanoparticles used, in three main classes: nanocomposites containing magnetic nanoparticles, metal nanoparticles, and carbon based nanostructured fillers.

### 3.1. Nanocomposites Containing Magnetic Nanoparticles

**Figure 5:**
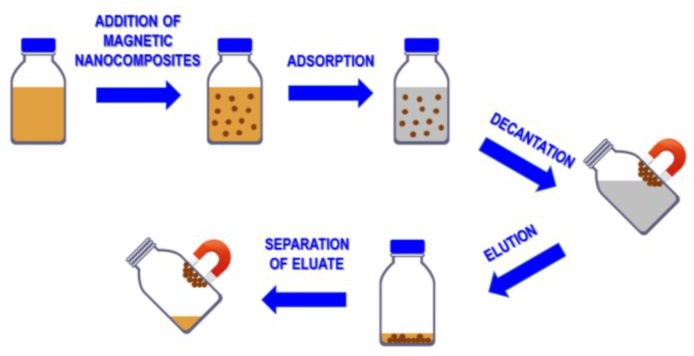
Simplified scheme showing the different steps of the magnetic solid phase extraction process.

Polymer based materials with high absorption properties can be usefully modified, imparting them magnetic properties. This strategy can be very useful to apply them for magnetic separation in several industrial sectors [74,75] recovering spent sorbents at the end of the process. Magnetic haemocompatible adsorbents were also proposed for extracorporeal detoxification of blood [76]. A schematic illustration of the magnetic solid phase extraction (MSPE) is reported in Figure 5 [77]. First, the magnetic adsorbent is added to the solution containing the substance to be removed. After a certain period, that depends on the adsorption kinetic of the magnetic sorbent, the analyte is adsorbed by the magnetic adsorbent. This latter is easily separated from the solution by application of an external magnetic field. During this stage, the solution is removed, and finally the sorbent is washed and regenerated by treatment with a proper solution.

**Figure 6:**
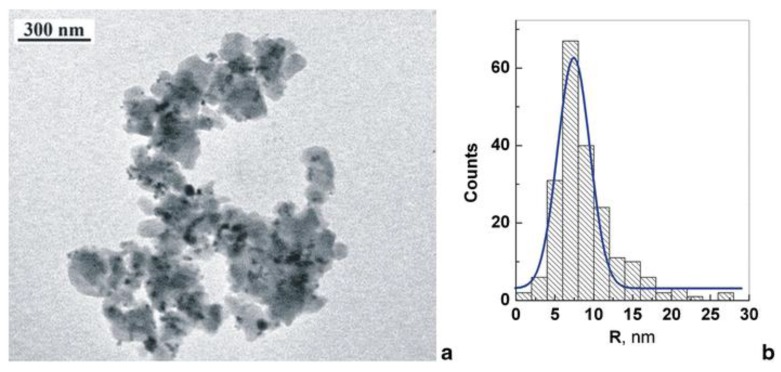
(**a**) TEM micrograph of Fe_3_O_4_ nanoparticles embedded in a fragment of commercial hyper-crosslinked polystyrene MN200 (Purolite Int., UK); (**b**) histogram and differential function of magnetite nanoparticles size distribution (200 particles from 12 nanocomposite fragments). Reprinted by permission from Springer (Berlin, Germany) [76], copyright 2014.

Magnetite (Fe_3_O_4_) and, less frequently, maghemite (γ-Fe_2_O_3_) nanoparticles are used to impart permanent magnetic properties to sorbents. In particular, magnetite nanoparticles with controlled shape and size can be obtained via the chemical co-precipitation method, starting from Fe(II) and Fe(III) water solutions with molar ratio Fe(II)/Fe(III) = 2.45 [78]. By simple in situ synthesis of magnetic nanoparticles in the presence of various HCL polystyrenes with a specific surface area of about 1000 m^2^/g, Pastukhov et al. [76] were able to incorporate 12–14 wt % of Fe_3_O_4_ in mesoporous polystyrene beads, without significantly affecting their porosity and adsorption properties. The final amount of iron oxide nanoparticles embedded, the nanoparticle size and properties of the final nanocomposites were found dependent on the concentration of iron salts water solutions and on after-treatment procedures. In particular, Fe_3_O_4_ nanoparticles with an average size of about 2 nm were obtained in a microporous HCL polystyrene obtained from ST/DVB gel-type copolymer, whereas larger nanoparticles with an average size of about 7 nm were obtained in beads of commercial biporous HCL MN200 (Purolite Int., Llantrisant, UK) having micro- and macropores with radii of ~0.7 nm and ~50 nm, respectively. TEM micrograph and size distribution of magnetite nanoparticles that are embedded in the MN200 resin is reported in Figure 6. The obtained materials were successfully tested for sorption of various toxic substances, such as iron pentacarbonyl, cyclopentadiene mangan tricarbonyl, chromium pentacarbonyl, and chloropicrin.

With a different approach, Zhou et al. [79] embedded preformed Fe_3_O_4_ nanoparticles that are coated with oleic acid by suspension polymerization of DVB, followed by a hyper-crosslinking step. They showed an improved tetracycline adsorption capacity of the obtained magnetic HCL resins over commercial resins. Furthermore, they systematically studied the effects of SSA and pore size distribution on tetracycline adsorption [80], showing that larger SSA induces the presence of more adsorption sites and promotes a higher adsorption capacity and an improved adsorption behavior. Moreover, larger pores reduce the diffusion resistance in the resin channels and induce faster adsorption kinetics. On the contrary, narrow pores lead to the accumulation of adsorbed molecules in the channels, causing pore obstruction and inducing poor resin reusability. As indicated, in these systems, nanoparticles were coated with oleic acid to promote nanoparticle dispersions within the polymer. Other substances, such as silanes, were also used as coupling agents [81]. The obtained HCL nanocomposite materials, characterized by SSA close to 1150 m^2^/g, an average pore size 3.3 nm, and mesopore/micropore volume ratio of about 3.7, showed fast adsorption kinetics and a large adsorption capacity towards nitrofurazone (180 mg/g) and oxytetracycline (200 mg/g). For these systems, the reusability was also investigated, evaluating the decrease of their adsorption capability toward tetracycline after several adsorption/desorption cycles. Results showed that nanocomposites that were obtained by resins with wide pore structure (average pore size 8.4 nm) had only a 4.94% decrease in adsorption efficiency after ten reuses, whereas nanocomposites that were obtained by resins with a narrow pore size (3.3 nm) displayed a significant decrease, about 23% in adsorption capacity, after the same number of cycles. This phenomenon was ascribed to accumulation of target molecules in small pores that caused pore blockage.

Tolmacheva et al. [82,83] obtained HCL resins loaded with preformed magnetite nanoparticles by simple stirring the commercial HCL polystyrene Diapak P-3 (BioKhimMak ST), previously dissolved in methanol, with the nanoparticles in an electromechanical vibromixer for 40 min. The so obtained magnetic adsorbent was then separated by the application of an external magnetic field, washed, and dried. Through this method, magnetic nanocomposite sorbents containing up to 30 wt % of magnetite were obtained. Even at the highest nanofillers loadings, the adsorption capability of the nanocomposite towards oxytetracycline was negligibly affected. Nevertheless, increasing the relative amount of nanofiller, a gradual detrimental effect on the degree of desorption of oxytetracycline using a methanol/acetonitrile solution was recorded. Using the same conditions, the system containing 5 wt % of magnetite showed a degree of desorption of 95%, whereas this value decreased to 50% for the sample containing 30 wt % of magnetite. This phenomenon was attributed to the oxytetracycline/magnetite interactions [82]. The same materials were also tested for the removal of sulfonamides from river water and milk samples, showing a recovery efficiency >85% [83].

In general, the approach based on the in-situ synthesis of magnetic nanoparticles showed a better distribution of the nanoparticles within the porous structure of the resins, with no relevant agglomeration phenomena evidenced. This is clearly shown in Figure 5. On the contrary, the ex-situ approach revealed in some cases agglomeration phenomena of Fe_3_O_4_ nanoparticles on the surface of the resin beads [77]. For what concerns the large-scale applicability of these systems, even if some results were reported on their reusability and fouling resistance [80], their long-term efficiency still needs a deeper evaluation.

Moreover, recently, Li et al. [84] developed magnetic polystyrene/ethylene diamine tetraacetic acid (PS/EDTA) magnetic microsphere. The maximum adsorption capacity of these systems towards tetracycline was estimated at 166 mg/g. Even if the obtained materials are not based on HCL PS, they are to be mentioned because, in this case, magnetic Fe_3_O_4_ nanoparticles were embedded in preformed PS/EDTA microspheres by direct mixing with ferric ammonium oxalate followed by a solvothermal treatment, an approach still unexplored for polystyrene based porous systems.

### 3.2. Nanocomposites Containing Metal Nanoparticles

Metallic nanoparticles supported onto inorganic or carbon-based materials are widely used as heterogeneous catalysts in many industrial sectors, including chemical manufacturing and energy-related applications [85]. Nevertheless, catalysts that are immobilized on these supports often show poor stability as their catalytic activity is reduced by aggregation and leaching phenomena. The use of high SSA materials, such as hyper-crosslinked polymers, can represent a valid alternative to inorganic and carbon-based materials as supports for various metal nanoparticles, allowing for the realization of microheterogeneous catalysts that combine the advantages of both homo- and heterogeneous systems and are suitable for various catalytic applications [86].

The first studies on the possible use of nanocavities typical of hyper-crosslinked polystyrenes as nanoreactors are due to Sidorov et al. [87], who grew the Co nanoparticle by the impregnation of ST/DVB resins with Co organometallic solutions, followed by drying, hydrolysis, and thermolysis. By this method, a wide range of Co contents (2–8 wt %) and nanoparticle sizes of 1–3 nm, which are comparable to the average pore size of the polymeric matrix, were obtained. With a similar strategy, Pt nanoparticles were grown within the pores of the same microporous resin by impregnation with platinic acid solutions, followed by reduction of the obtained Pt(II) complexes. These materials were highly selective (up to 98% at 100% conversion) for the catalytic oxidation of l-sorbose to 2-keto-l-gulonic acid [88]. Further studies on Pt nanoparticles that are grown on commercial micro/macroporous polystyrene resins (Macronet MN 270/386, Purolite Int., UK), demonstrated that the presence of macropores can increase the catalytic activity of these systems by a factor 4.6, as large pores improve the accessibility of Pt nanoparticles to the reaction species [89]. A similar system with Pt nanoparticles in different forms (unmodified, reduced with various reagents, and impregnated with cinchonidine) was tested for the hydrogenation of ethylpyruvate, demonstrating its potential for the enantioselective catalysis of activated ketones hydrogenation [90]. A commercial PS resin containing from about 0.1 wt % up to about 5 wt % (evaluated by X-ray fluorescence analysis [91]) of mixed Pt-containing nanoparticles, was also proposed for water purification applications, and was tested for phenol catalytic wet air oxidation. Nanoparticles size that was obtained within the pores of the HCL resin measured about 2.1–2.3 nm, and the obtained Pt species included Pt(II), Pt(IV) and relatively lower amounts of Pt(0). The highest conversion, activity, and selectivity, markedly exceeding those of the conventional Al_2_O_3_-supported Pt catalysts, were obtained for the HCL resin containing 0.95 wt % Pt, while the catalyst containing the highest Pt amount was found the less active and selective. This result was explained when considering that nanoparticle agglomeration phenomena and pore blockage both contribute to the reduction of the catalytic activity of nanoparticles within micro/macroporous resins when the nanoparticle loading exceeds an optimal amount [92].

**Figure 7:**
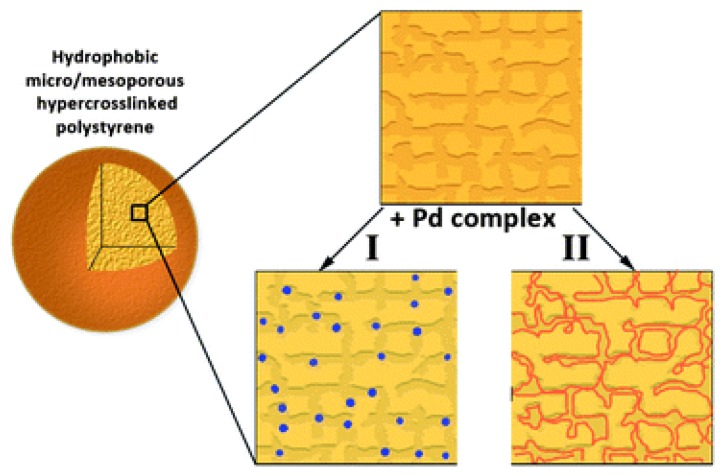
Schematic representation of Pd complexes embedded in HCL polystyrene depending on the complex/resin compatibility: I for low complex/resin compatibility; and, II for high compatibility. Reproduced from ref. [93] with permission of the Royal Society of Chemistry (London, UK).

A subsequent work of the same research group [93] was focused on the growth of Pd complexes within the pores of HCL polystyrenes. They demonstrated that by varying the organic components of the Pd complex, different morphologies could be obtained within the pores of the resins based on the compatibility between the resin and the complex. Large nanoparticles were obtained for the bis-acetonitrile/Pd complex (about 5 nm diameter), whereas no nanoparticles were obtained in the case of the highly compatible styrene/Pd complex (Figure 7). The obtained systems were tested as catalysts for the oxidation of D-glucose, revealing that best catalytic efficiency, in terms of conversion and selectivity, are obtained at intermediate complex/resin compatibility, when nanoparticles size ranges between about 4 and 2 nm. A further contribution on the understanding of the catalytic potentiality of Pd nanoparticles supported on HCL polystyrenes was given by Nikoshvili et al. [86], who evaluated the relevance of the solvent effect for these systems for the selective hydrogenation of 2-methyl-3-butyn-2-ol to 2-methyl-3-butene-2-ol. Recently, Pd nanoparticles that were supported on HCL polystyrenes were also tested as catalysts for stearic acid hydrodeoxygenation, revealing that the use of HCL polystyrene containing about 1 wt % Pd allows for converting stearic acid with up to 97% heptadecane yield [94].

Also, Ru nanoparticles have been embedded in HCL polystyrenes by treatment of the resin with a Ru(OH)Cl_3_ solution, followed by treatment with NaOH and H_2_O_2_ [95]. Very small nanoparticles were obtained (mean diameter 0.5–0.8 nm), coexisting with less numerous larger nanoparticles (mean diameter up to 3.0 nm), and some aggregates. The latters are attributed to the hydrophobic nature of the polymer matrix, that prevents the homogeneous distribution of the metal during the impregnation of the resin. The obtained nanoparticles have a mixed valence structure, with both oxidic and metallic components. The catalytic activity and selectivity of the realized systems was tested towards the oxidation of d-glucose to d-gluconic acid, and the system containing 0.74 wt % of Ru was found to be the most performant with respect to systems containing 0.05 and 2.71 wt % of Ru. Other HCL resin systems containing Ru nanoparticles were tested as catalysts. Protsenko et al. [96] realized a nanocomposite system containing about 5 wt % Ru and found it comparable with commercial Ru/C systems for the selective hydrogenation of levulinic acid to γ-valerolactone, with yields >99%. A similar system was tested for the catalytic hydrogenation of d-glucose to d-sorbitol, revealing two routes for the reaction, one based on the interaction of d-glucose with the spilled-over hydrogen supplied by the catalyst, the other based on the interaction of the substrate with hydrogen coming from the reaction medium [97]. Moreover, with an interesting strategy, Ru-containing catalysts were also realized on HCL polystyrene resins, either unfunctionalized, or functionalized with amino and sulphate groups. The catalyst based on unfunctionalized resin and containing 1.0 wt % Ru was found the most active for cellulose hydrogenolysis to sorbitol and mannitol [98].

### 3.3. Nanocomposites Containing Carbon-Based Nanostructured Fillers

Nanostructured carbon materials, such as carbon nanotubes (CNT) and graphene, at first being mainly studied for their remarkable electronic, thermal, and mechanical properties [99], have soon been considered extremely attractive for their adsorption properties. Several researches have been focused on their adsorption properties, either for gas adsorption or for water purification [100,101,102,103,104,105]. Filters based on CNT have been realized for purification of contaminated drinking water, which are able to electrochemically oxidize organic contaminants, bacteria, and viruses [106]. Depending on synthetic parameters, different interesting applications of nanostructured carbons are exploited. Xhao et al. [107] prepared sponges by graphene oxide processing that were able to adsorb different dyes, oils, and organic solvents from water. Graphene and graphene oxide (GO) interactions with organic substances in aqueous dispersion were studied by many research groups, and methylene blue (MB) adsorption, in particular, was exploited to correlate the amount of methylene blue adsorbed to the GO specific surface area [108,109,110,111]. MB uptake was found to be increasing with increasing the oxidation degree of graphene oxide, while, for nano-graphene oxides that were prepared by a modified Hummers method, MB adsorption was found to be notably high, around 600–700 mg/g, almost indifferently from the oxidation level, probably due to the good stability of the nano-graphene oxide water dispersions, due to their reduced lateral size [112].

While considering the wide adsorption mechanisms of carbon-based nanostructured fillers, ranging from π–π interactions, hydrophobic effect, hydrogen bonds to electrostatic interactions [113], recently, in our research group, we investigated the interaction of such highly adsorbent materials with high surface area polymers as HCL styrene-based polymers. In particular, we prepared a new category of nanocomposites, based on a polymer phase made of DVB, ST and VBC and containing surface modified carbon nanotubes [114], graphene nanoplatelets (GNP), and surface modified graphene oxide [34], inspecting the structure, morphology, and adsorption properties of the obtained nanocomposites.

**Figure 8:**
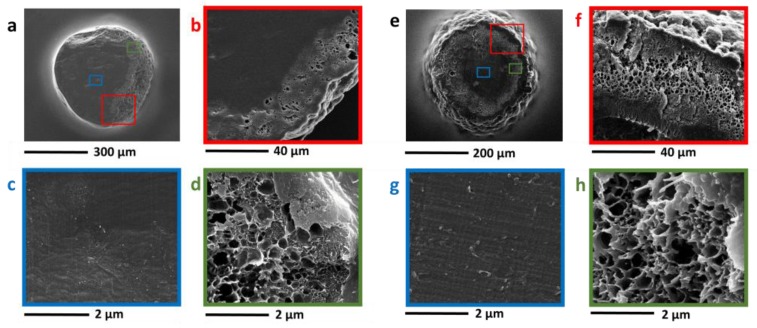
TEM micrographs of sections of the nanocomposite (**a**–**d**) DVB/VBC and (**e**–**h**) DVB/ ST/VBC beads containing 3 phr carbon nanotubes (CNT). Reproduced from Ref. [114] with permission from the Royal Society of Chemistry (London, UK).

In a first work, HCL polymers and nanocomposites based on DVB, ST, and VBC and containing multi-walled carbon nanotubes were prepared by a classical hyper-crosslinking two-step route, consisting in a suspension polymerization followed by Friedel-Crafts reaction [114]. The effect of CNT on nanocomposites with a different degree of hyper-crosslinking was explored by realizing polymers and nanocomposites that are composed of a polymeric matrix based on DVB, ST, and VBC in two different molar composition with a variable VBC content. In particular, a DVB/VBC system containing 2 mol % of DVB and 98 mol % of VBC, and a DVB/ST/VBC set of sample containing 2 mol % of DVB and 49 mol % of either ST and VBC, both containing up to 3 phr of CNT, were prepared. CNT were included in the monomeric matrices after proper surface grafting with poly(vinylbenzyl chloride) (PVBC) to promote the nanofiller inclusion and dispersion in the organic matrix, and to induce their participation to the hyper-crosslinking reaction. In fact, the functionalization strategy yields surface modified CNT with a layer of PVBC rich of unreacted chloromethyl groups that can be activated in the hyper-crosslinking step. CNT surface modification was effective to induce the inclusion of the nanofillers into the polymer beads, but, at higher loadings, the selective localization of the nanofiller became a relevant phenomenon (see Figure 8).

The surface area analysis of the HCL polymers and nanocomposites revealed that the carbon nanotubes inclusion had a significant effect on the pore size distribution of the nanocomposites, leading to higher microporous fractions in the nanocomposites containing 1.5 phr of CNT. At this composition, both the DVB/VBC and the DVB/ST/VBC systems showed a higher adsorption capacity towards H_2_, higher CO_2_/N_2_ selectivity, and phenol adsorption from aqueous solution, proving that the CNT inclusion in Davankov’s type HCL resins can be a promising approach to modulate and improve their adsorption properties.

**Figure 9:**
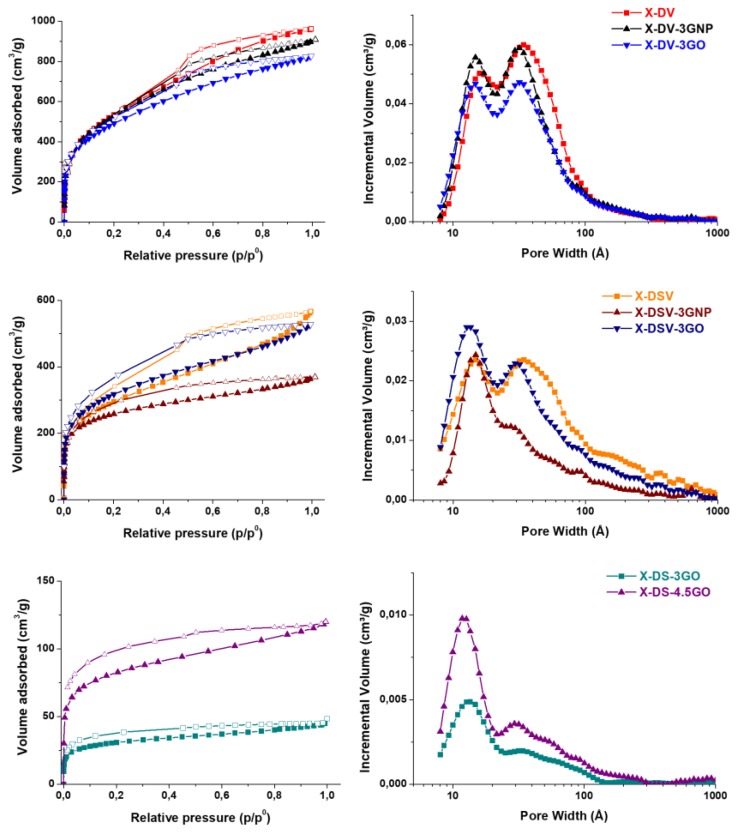
Isotherms of adsorptions (left) and density functional theory pore size distributions (right) of the DVB/VBC, DVB/ST/VBC and DVB/ST systems. The DVB/VBC based hyper-crosslinked polymer (HCP) is coded X-DV, the DVB/ST/VBC based HCP is coded X-DSV and the DVB/ST based HCP is coded X-DS. The nanocomposites containing graphene nanoplatelets (GNP) or surface modified graphene oxide (GO) are coded as the corresponding polymeric samples adding a suffix indicating the amount (phr) and the type of nanofiller. Reprinted with permission from [34]. Copyright 2017 American Chemical Society (Washington, United States of America).

Moreover, in a following work [34], moved by the need of a new efficient synthetic approach for the realization of HCL nanocomposites with high SSA, we set up a different route to prepare HCL styrene-based nanocomposites containing nanostructured carbon materials. In particular, the suspension step of the traditional procedure was replaced by a bulk polymerization step, which permitted the better dispersion of the nanofillers and to obtain new interesting structures. The proposed procedure also allowed for obtaining significantly higher yields and reducing the amount of waste chemicals. All of the obtained nanocomposites based on DVB/VBC or DVB/ST/VBC matrices, containing 3 phr of GNP or PVBC-surface modified GO presented uniform dispersion of the nanofillers and improved adsorption properties. The microporosity fraction of the HCL nanocomposites significantly increased in both systems with the inclusion of GNP or GO, as evident from the adsorption isotherms and the density functional theory (DFT) pore size distribution curves, as displayed in Figure 9.

**Figure 10:**
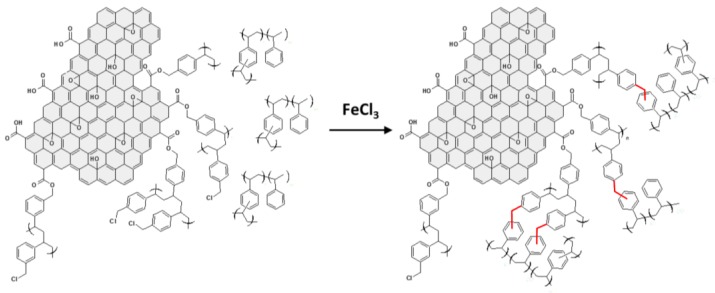
Schematic representation of the hyper-crosslinking reaction at the interface between PVBC-grafted GO and poly(DVB-ST). Crosslinking derived from the hyper-crosslinking reaction are evidenced in red. Reprinted with permission from [34]. Copyright 2017 American Chemical Society (Washington, United States of America).

Particularly interesting, in this work, was the development a new type of HCL nanocomposites without an internal crosslinker monomer, in which the hyper-crosslinking was only due to the surface modified GO. HCL nanocomposites that were based on a DVB/ST matrix containing up to 4.5 phr of PVBC-modified GO displayed SSA up to ~300 m^2^/g. These materials show a new kind of porosity, only developed at the nanofiller/organic matrix interface. The mechanism for their realization is schematically represented in Figure 10. PVBC-modified GO reacts with the precursor polymer poly(DVB-ST), and crosslinks are formed between the chloromethyl groups of the grafted PVBC layer and the aromatic rings of the poly(DVB-ST) matrix.

These nanocomposites show a porosity distribution tending towards a unimodal distribution, with a major peak around 12 Å (samples coded X-DS-3GO and X-DS-4.5GO in Figure 9). The specific surface area and pore structure of these nanocomposites are very interesting, especially if considering the chlorine content of the corresponding precursors, which is around 0.10 wt %, to be compared with 22.8 and 13.5 wt %, respectively, of the DVB/VBC and DVB/ST/VBC systems. 

These kind of nanocomposites were used as functional fillers in porous membranes and hydrogels for water remediation. In particular, HCL polymers and nanocomposites were dispersed in polysulfone porous membranes, and these systems were tested for phenol removal from water solution. The HCL particles induced a significant increase in the phenol uptake with respect to neat polysulfone membrane [34]. Moreover, in the systems containing graphene oxide, the GO oxygen containing groups, under certain conditions, led to the formation of active oxygen species, such as hydroxyl radicals. These active species could promote photocatalytic activity of the material for possible applications in water remediation [115,116]. Furthermore, composite hydrogels that were based on chitosan and filled with hyper-crosslinked polymer particles were recently realized, showing broad-spectrum adsorption towards different organic compounds [117]. Adsorption experiments revealed a synergistic effect between chitosan and hyper-crosslinked PS-based particles, as the composite hydrogels were able to remove both anionic and cationic dyes.

## 4. Concluding Remarks

Hyper-crosslinked polymers group various classes of macromolecular systems that are realized by extensive crosslinking of a linear or lightly crosslinked precursor polymer. Among them, hyper-crosslinked polystyrenes show outstanding properties, including high specific surface area and an extraordinary adsorption capability. Several strategies have been recently developed to tailor functional properties of these materials for specific applications. In particular, new synthetic routes have been explored to modulate their mesoporosity/microporosity ratio, to insert functional groups in order to improve their gas sorption capacity and selectivity, and to optimize their properties for their application in water remediation, exploiting their ability in sequestering organic pollutants. Moreover, a large effort has been carried out to tailor the morphology of the realized systems (monoliths, microparticles, nanoparticles, membranes), in this way contributing to enlarging their suitability in different fields.

As soon as discovered, the microporous structure of hyper-crosslinked polystyrenes was also considered potentially interesting to guest nanoparticles. Currently, there are several on-going researches that are aimed at tailoring functional properties of these micro/mesoporous systems by embedding different nanoparticles within the nanocavities of the polymer. These works are either based on the in situ post-growth of nanoparticles on the hyper-crosslinked PS matrix, either on the direct embedding of pre-formed nanoparticles during the pre-polymerization step, followed by hyper-crosslinking. Different classes of nanostructured fillers have been tested. 

In particular, several hyper-crosslinked nanocomposites containing magnetic iron oxide nanoparticles have been successfully realized, characterized by permanent magnetic properties, and tested in magnetic solid phase extraction. Other hyper-crosslinked polystyrene-based nanocomposite systems containing metal and mixed metal/metal oxide systems (mainly containing Pt, Pd, and Ru) have been reported as very efficient and selective catalytic systems, in which the high specific surface area hyper-crosslinked resin is used as a support for metal based catalysts. 

More recently, hyper-crosslinked polystyrene-based nanocomposites containing carbon based nanostructured fillers (multi-walled carbon nanotubes, graphene nanoplatelets, and graphene oxide) were also reported, in which nanofillers were found able to modulate the micro/mesoporosity of the resin and its gas sorption selectivity. Within this class of nanocomposites, very interesting was the development a new type of hyper-crosslinked systems, in which the microporosity is only generated at the interface between surface functionalized nanofillers and the organic matrix. 

Even if a large literature on this subject is already available, there is still space to investigate and optimize the structure and properties of this class of materials for specific applications. In particular, recent developments of easy synthetic routes to realize hyper-crosslinked nanocomposites offer unexplored possibilities to impart new functional properties to hyper-crosslinked polystyrenes.
